# Chondroitin sulfate-decorated cupper-benzene dicarboxylate framework as an efficient passive and active targeting nanomedicine for anticancer methotrexate delivery

**DOI:** 10.1016/j.ijpx.2025.100403

**Published:** 2025-09-25

**Authors:** Siamak Javanbakht, Reza Mohammadi

**Affiliations:** Polymer Research Laboratory, Department of Organic and Biochemistry, Faculty of Chemistry, University of Tabriz, Tabriz, Iran

**Keywords:** Chondroitin sulfate, Drug delivery, Metal-organic framework, Methotrexate, Receptor

## Abstract

In this research, an advanced drug delivery system was developed by decorating the copper-benzene dicarboxylate framework (Cu(BDC)) with the multifunctional chondroitin sulfate (ChS), termed Cu(BDC)/ChS. This novel system is designed for both active and passive targeting, featuring a pH-sensitive release mechanism that enhances drug effectiveness. Different characterization techniques confirmed the successful synthesis of the Cu(BDC)/ChS nanocomposite. In-vitro experiments evaluating the loading and release of methotrexate (MTX) showed that the release rate was significantly higher at pH 4.5, releasing 70 % over 92 h at 41 °C, in contrast to less than 20 % at pH 7.4 at 37 °C. This pH responsiveness of the Cu(BDC)/ChS promotes drug release in environments alike to tumor tissues. Additionally, cytotoxicity tests revealed that MTX-loaded Cu(BDC)/ChS exhibited considerable cytotoxic effects on MCF-7 cancer cells, with IC50 value of ∼250 μg/mL after 48 h, accompanied by an increase in apoptosis rates. Remarkably, the overexpression of CD44 receptors on cancer cell surfaces underscores the significance of ChS-functionalized systems in promoting selective cancer cell apoptosis, while exhibiting minimal cytotoxicity toward normal HUVEC cells. Overall, the findings indicate that the combination of Cu(BDC) and ChS holds promise for developing effective platforms for anticancer drug delivery.

## Graphical abstract

**Figure:**
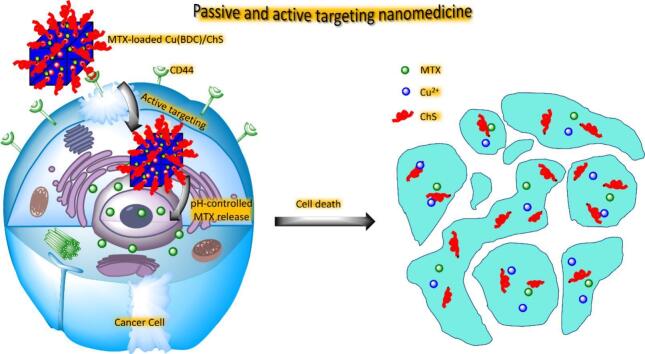

## 1 Introduction

Cancer remains one of the foremost causes of death globally, resulting in millions of fatalities annually (Liu et al., 2020). The prevalence of cancer is projected to rise significantly, with estimates suggesting that by 2040, there could be around 28 million new cases each year. This complex disease has garnered extensive attention from the medical research community for many years (Ma et al., 2022; Seyfried and Shelton, 2010). Traditional treatment modalities for cancer primarily include surgery (Li et al., 2020), radiation therapy, and chemotherapy (Dehnoee et al., 2023), all aimed at destroying or diminishing cancerous cells (Hassan et al., 2010). However, these approaches often come with considerable drawbacks, including the emergence of drug resistance in collateral damage and chemotherapy to healthy tissues during radiation treatment (Zafar et al., 2025). To mitigate these adverse effects, targeted drug delivery systems (DDSs) have been developed as a great solution (Manish and Vimukta, 2011; Mohammadzadeh et al., 2024b), focusing on the precise delivery of therapeutic agents to tumor tissues while ensuring controlled release (Jain et al., 2015; Mohammadzadeh et al., 2024a).

Recent advancements have introduced a variety of innovative techniques and materials, particularly at the nanoscale, that exhibit unique properties suitable for drug delivery applications (Ahmeda et al., 2020; Bai et al., 2021). Coordination polymers, metal-organic frameworks (MOFs), have gained prominence in this field due to their distinctive characteristics (Nezhad-Mokhtari et al., 2023; Poursadegh et al., 2024a). MOFs are porous materials composed of metal ions/clusters linked by organic ligands (Kitagawa, 2014; Zhou and Jeffrey, 2012). Their tunable pore sizes, high surface area, pH sensitivity, and diverse chemical functionalities position them as excellent candidates for DDSs (Giménez-Marqués et al., 2016; Moharramnejad et al., 2023). These frameworks can be engineered for encapsulation, storage, and targeted release of therapeutic agents, thereby enhancing the efficacy and longevity of drugs while minimizing side effects (Gu et al., 2024; Kateb et al., 2011).

CD44 is a cell surface receptor that is frequently overexpressed in various cancer types, including pancreatic, lung, ovarian, and breast cancers (Le et al., 2021; Liu et al., 2018). This overexpression presents an opportunity for developing targeted cancer therapies (Wang et al., 2018). Chondroitin sulfate (ChS), a polymer structurally similar to heparin and hyaluronic acid, specifically targets CD44 receptors (Li et al., 2014; Tran et al., 2014). This targeting capability enables the efficient internalization of nanoparticles by tumor cells through CD44-mediated mechanisms (Aqil et al., 2017). In addition to its targeting potential, ChS is highly regarded in drug delivery applications due to its excellent biocompatibility, stability, bioactivity, and biodegradability (Li et al., 2019; Zhu et al., 2015). Studies have demonstrated that ChS-coated carrier can effectively deliver doxorubicin and curcumin to breast cancer cells, resulting in improved inhibition of breast tumor metastasis (Azimijou et al., 2024; Fernandes et al., 2018; Mohammadi et al., 2024).

**Fig. 1:**
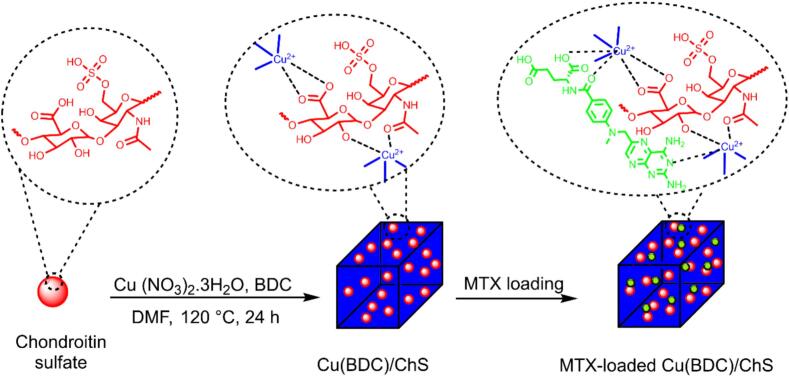
Schematic representation of the Cu(BDC)/ChS nanomedicine synthesis and possible MTX loading interactions.

Our research team has recently focused on combining various MOF structures with saccharides to create new drug carriers (Darvishi et al., 2024; Hamedi et al., 2024; Poursadegh et al., 2024a). To our knowledge, there has been no prior investigation into copper benzene-1,4-dicarboxylate framework, Cu(BDC),-based MOF modified with ChS for such applications. Therefore, this study aims to explore a novel nanocomposite designed for both active and passive targeting in DDSs. Based on existing literature highlighting the pH-sensitive properties and positive charge of Cu(BDC) MOFs (Abbasian and Khayyatalimohammadi, 2024; Cun et al., 2022), we have chosen to modify these frameworks with ChS to leverage their advantageous features (Fig. 1), such as pH responsiveness, porous architecture, drug loading capacity, degradation in acidic environments, and affinity for cellular receptors. The modified MOFs will be evaluated for their potential in delivering methotrexate (MTX, a model anticancer drug) effectively through targeted mechanisms.

Numerous DDSs have been developed for MTX and other chemotherapeutics, including liposomes, polymeric micelles, dendrimers, carbon-based nanomaterials, and MOFs (Mukhtar et al., 2023). Although liposomes and polymeric carriers offer biocompatibility, their limitations in drug retention and tumor selectivity have been widely reported (Saad et al., 2008). MOF-based DDSs, such as ZIF-8 and MIL-101, have demonstrated promise due to their tunable porosity and stability (Vahdatkhah et al., 2025). However, most lack effective targeting ligands and exhibit limited pH-responsive behavior. In contrast, the Cu(BDC)/ChS nanocomposite presented here combines a MOF with the tumor-targeting capability of ChS, enabling both active (via CD44 receptor) and passive (via pH-sensitivity) targeting with controlled release properties. This dual-functional system is thus expected to improve therapeutic efficacy and reduce off-target cytotoxicity.

## 2 Experimental

### 2.1 Materials

Chondroitin sulfate, ChS, with an average molecular weight of 30,000 Da was sourced from Farabi Pharmaceutical Co., Iran. Terephthalic acid (benzene-1,4-dicarboxylic acid, BDC, 98 % purity), Copper(II) nitrate trihydrate (Cu(NO_3_)_2_·3H_2_O, ≥99 % purity), and dimethylformamide (DMF, 99.8 % purity) were obtained from Merck Co. Methotrexate (MTX) was procured from Zahravi Pharma Co., Iran.

### 2.2 Synthesis of Cu(BDC)/ChS

According to the reported method with some modification (Darvishi et al., 2024), to synthesize the Cu(BDC)/ChS composite, ChS (0.20 g) was dissolved in DMF (40 mL) for approximately 20 min to create a uniform solution. Next, copper(II) nitrate trihydrate (0.48 g, 2 mmol) was added, followed by stirring for an additional 15 min. Afterward, BDC (0.32 g, 2 mmol) was introduced, and stirring continued for another 15 min. Then, the mixture was refluxed at 120 °C for 24 h. Upon cooling, the resultant solid was collected through filtration, washed several times with DMF, and allowed to dry at ambient temperature, yielding approximately 0.69 ± 0.05 g of product.

### 2.3 Characterization

To confirm the successful synthesis of the nanocomposite, various characterization techniques were employed. Fourier-transform infrared spectroscopy (FT-IR) was conducted using a Bruker Instruments Aquinox 55 spectrophotometer using KBr pellets. X-ray diffraction (XRD) analysis was performed with a Siemens D500 instrument at 35 kV utilizing Cu-Kα radiation. Ultraviolet-visible spectroscopy (UV–Vis) measurements were taken using a Shimadzu Model 1700 spectrophotometer. Thermogravimetric analysis (TGA) was executed under an oxygen atmosphere at a heating rate of 10 °C/min using a LINSEIS STA PT-1000 apparatus from Germany. Scanning electron microscopy (SEM) combined with energy-dispersive X-ray spectroscopy (EDS) was carried out using a TESCAN MIRA3-FEG SEM system. For surface morphology analysis, transmission electron microscopy (TEM) was performed with a Philips EM 208S instrument. Zeta potential (ZP) and dynamic light scattering (DLS) assessments were conducted using a Malvern model MAL1032660 particle sizer.

### 2.4 MTX loading

For drug loading, Cu(BDC)/ChS (50 mg) was immersed into 50 mL of a stock solution of MTX (100 ppm) and stirred for 48 h at ambient temperature in the dark. Following this period, the precipitate was centrifuged and washed with deionized water to eliminate any unloaded MTX. The amount of MTX loaded onto the nanocomposite was quantified using UV–Vis spectroscopy based on a calibration curve for MTX. The loading efficiency (LE) was determined with the following Eq. (1).

(1) $$\%\mathrm{LE}=\frac{M\mathrm{ass}\;\text{of drug in nanomedicine}}{\mathrm{Mass of drug}\;\mathrm{fed}\;\mathrm{initially}}\times 100$$

### 2.5 In vitro MTX release

The drug release profile from the MTX-loaded Cu(BDC)/ChS nanomedicine was evaluated in-vitro at pH 4.5 and pH 7.4. Typically, each sample weighing around 10 mg was placed in a PBS buffer solution at these pH levels, mimicking tumor conditions at pH 5 and physiological conditions at pH 7.4, at temperatures of 41 °C and 37 °C, respectively. At predetermined time intervals, 2 mL of supernatant was withdrawn from the release medium and replaced with fresh buffer solution. The concentration of released MTX was measured using UV–Vis spectrophotometry, and the percentage of drug released was calculated using Eq. (2):

(2) $$\mathrm{Drug release}=\frac{\mathrm{the amount of released drug}\;}{\mathrm{the amount of loaded drug}}\times 100$$

### 2.6 Kinetics of drug release

The MTX release kinetics from the MTX-loaded Cu(BDC)/ChS nanomedicine were analyzed considering different mathematical models, such as, zero-order, first-order, Weibull, Higuchi, and Korsmeyer-Peppas models through Mathcad version 15 software (Javanbakht et al., 2020; Luo et al., 2019).

### 2.7 Cytotoxicity study

The cytotoxic effects of the synthesized materials were assessed in vitro using MTT assays on human breast cancer cell line (MCF-7) and human umbilical vein endothelial cells (HUVEC). The cells were seeded in 96-well plates at a density of 6000 cells/well in 200 μL cell medium and incubated for one day (Yang et al., 2016). Following incubation, cells were treated with MTX, MTX-loaded ChS, and MTX-loaded Cu(BDC)/ChS at equivalent concentrations while blank Cu(BDC) and Cu(BDC)/ChS samples served as controls to evaluate cytocompatibility. After incubating for an additional 48 h, cell viability was measured by changing the culture medium with a mixture of MTT solution (3 mg/mL in fresh culture medium). After 4 h, to dissolve the formazan crystals formed by viable cells, DMSO was added at 37 °C, and cell viability was quantified using a multi-well plate reader at 570 nm (Quant Bio-Tek Instruments, Winooski, VT, USA).

### 2.8 DAPI staining

DAPI staining was utilized to detect nuclear fragmentation and condensation in apoptotic cells induced by treatment with samples at their respective IC50 values over a period of 48 h on glass coverslips seeded with MCF-7 cells (5 × 10^5^ cells/well). After treatment, the cells were washed, fixed using paraformaldehyde, permeabilized with Triton X-100, stained with DAPI, and subsequently examined for apoptosis using fluorescence microscopy.

### 2.9 Statistical analysis

All experiments were performed in triplicate, and data are expressed as mean ± standard deviation (SD). Statistical significance among groups was determined using one-way analysis of variance (ANOVA), performed in Microsoft Excel software with significance set at a *P* value less than 0.05; all experiments were conducted in triplicate to ensure reliability.

## 3 Results and discussion

### 3.1 Nanomedicine characterization

**Fig. 2:**
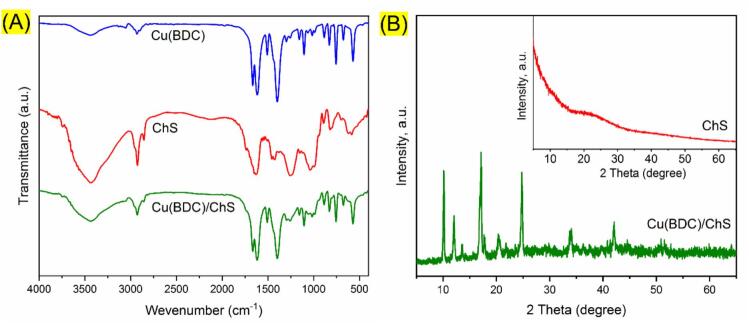
A) The FT-IR spectra of ChS, Cu(BDC) and Cu(BDC)/ChS. B) The XRD pattern of ChS and Cu(BDC)/ChS.

FT-IR spectroscopy was employed to analyze the functional groups and structural characteristics of the synthesized materials. The FT-IR spectra for Cu(BDC), ChS, and the Cu(BDC)/ChS composite are depicted in Fig. 2A. In the spectrum of Cu(BDC), peaks at 3442, 2922, 1506, 1620, 1398, and 754 cm^−1^ correspond to various vibrational modes: hydroxyl groups, C—H bonds, C=C bonds in phenyl rings of the ligand, symmetric and asymmetric stretching of carboxylic groups, and Cu—O stretching, respectively (Tari et al., 2016; Wang et al., 2020). The ChS spectrum exhibited peaks at 3442, 2927, 1626, 1560, 1247, 1039, and 850 cm^−1^, which are associated with the hydroxyl groups, aliphatic C—H bonds, C=O stretching, N—H bending, asymmetric and symmetric stretching of S=O bonds, and C-O-S bending vibrations, respectively (Azimijou et al., 2024). The FT-IR spectrum of the Cu(BDC)/ChS nanocomposite showed similar peaks to those of Cu(BDC), with slight variations in the S=O and C=O stretching region indicating successful grafting of ChS onto the Cu(BDC) framework.

XRD analysis was conducted to evaluate the crystalline structure of the synthesized nanocomposite. The XRD patterns for ChS and Cu(BDC)/ChS within a 2θ range of 5–55° are presented in Fig. 2B. ChS exhibited a broad peak around 22°, indicative of its amorphous nature (Harris et al., 2010). In contrast, the Cu(BDC)/ChS pattern revealed peaks characteristic of Cu(BDC) at 10.32°, 12.22°, 17.22°, 20.47°, 24.98°, 34.12°, and 42.22°, corresponding to specific planes (111), (200), (220), (222), (331), (402), and (420) that confirm its face-centered cubic (FCC) crystal structure (Carson et al., 2009; Javanbakht et al., 2018). On the other hand, slight shifts compared to the reported Cu(BDC) pattern can be observed, likely due to modification with ChS, confirming that the crystalline integrity of Cu(BDC) is maintained.

**Fig. 3:**
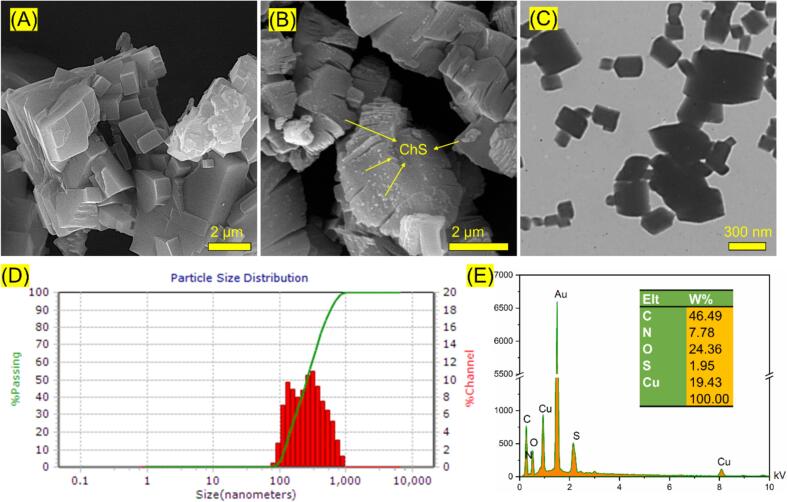
A) FESEM images of ChS and Cu(BDC)/ChS. B) TEM image of Cu(BDC)/ChS. The EDS (D) and particle size distribution (E) of Cu(BDC)/ChS.

The SEM images of Cu(BDC), Fig. 3A, displayed a cubic structure with smooth surfaces, affirming its crystalline nature (Abdollahi et al., 2022). The SEM image also indicated that cubic Cu(BDC) particles were surrounded by amorphous ChS particles, Fig. 3B, suggesting successful modification. TEM analysis further confirmed the cubic crystal structure attributed to Cu(BDC), Fig. 3C (Salama et al., 2018). Moreover, EDS analysis demonstrated the existence of C, O, N, S, and Cu elements in the structure of nanocomposite (Fig. 3E). Particle size distribution and zeta potential measurements were performed in deionized water; the average particle size for Cu(BDC)/ChS was approximately 305 nm with a polydispersity index (PDI) of 2.95 (Fig. 3D). Additionally, a positive zeta potential of +27.4 mV was recorded attributed to uncoordinated metal sites within the Cu(BDC) structure. This positive charge enhances colloidal stability over time in distilled water and suggests a greater possibility for interaction with negatively charged cancer cells (Javanbakht et al., 2019).

**Fig. 4:**
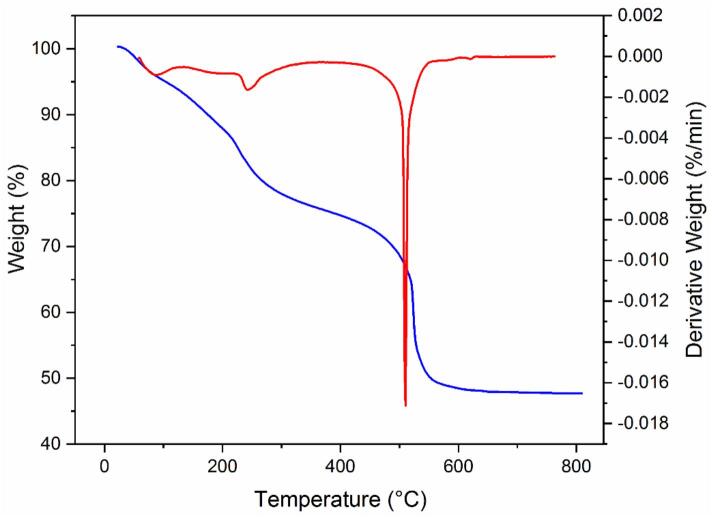
The thermogram of Cu(BDC)/ChS.

The TGA analysis was conducted to evaluate the composition of the synthesized Cu(BDC)/ChS as illustrated in Fig. 4. It observed three distinct weight loss stages: initial removal of adsorbed water between 25 and 150 °C; hydrolysis of ChS (Barkat et al., 2020) along with removal of DMF and dehydroxylation of copper clusters from 150 to 450 °C (Yang et al., 2018); and finally decomposition starting at about 500 °C due to combustion of organic linkers within the composite structure (Javanbakht et al., 2021). This analysis indicated that approximately 35 % by weight of ChS is present in relation to Cu(BDC).

### 3.2 In-vitro MTX loading and release

The loading efficiency of MTX in Cu(BDC)/ChS nanocomposites was calculated to be 70.8 ± 0.5 %, respectively, as measured by UV–Vis spectroscopy. The desired loading capacity of the Cu(BDC)/ChS nanocomposite over 48 h can be attributed to potential electrostatic and hydrogen bonding interactions between MTX molecules and ChS, along with host-guest and π-π stacking interactions with Cu(BDC) framework (Nezhad-Mokhtari et al., 2019).

**Fig. 5:**
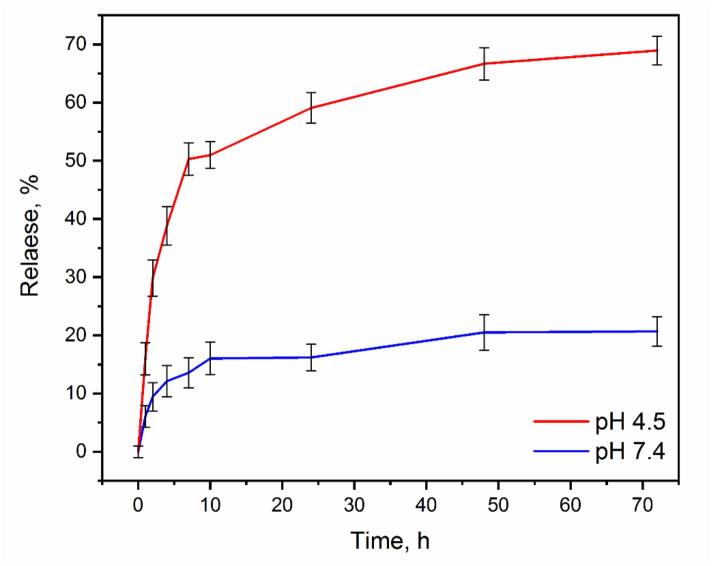
The MTX release profile of Cu(BDC)/ChS at pH 7.4 and 4.5. Statistically significant differences were determined using one-way ANOVA (*n* = 3, *P* < 0.05 between pH 4.5 and 7.4 at 72 h).

Fig. 5 illustrates the release profiles of MTX at two pH levels (7.4 and 4.5) from Cu(BDC)/ChS. Most of the MTX was released in acidic conditions (pH 4.5); however, a lower release rate was observed at pH 7.4. Notably, it showed a low initial burst release rate during the first 10 h and followed by a sustained release profile over 72 h. This increased release at pH 5 can be explained by the sensitivity of Cu(BDC)/ChS to acidic environments, which may lead to structural degradation of the Cu(BDC) and subsequent release of MTX (Darvishi et al., 2024). At pH 7.4, H bonding among different functional groups in ChS (i.e., COOH, SO_3_H, O—H) and the N—H and O—H groups of MTX remains stable. Conversely, at pH 5, protonation of the amino groups on MTX disrupts these hydrogen bonds, facilitating rapid release in acidic conditions. These findings indicate that the Cu(BDC)/ChS nanocomposite is a suitable candidate for passive anticancer drug delivery (Mohammadzadeh et al., 2024b; Poursadegh et al., 2024b).

Kinetic analysis revealed that the Weibull model best fits the release profiles for both Cu(BDC)/ChS nanocarriers (Table 1), suggesting that the structural characteristics of the nanocarrier play a more significant role than pH variations. Moreover, the Korsmeyer-Peppas model was applied to understand the release mechanisms better. The n values obtained for both pH were < 0.45, confirming that the mechanism of drug release predominantly follows Fickian diffusion (Contri et al., 2022), Table 1.

**Table 1:** The parameters of MTX release kinetics.

Kinetics models	Equations	Parameters	pH	
			7.4	4.5
Zero-order	F = K_0_t	R^2^	0.605	0.527
First-order	Ln (1-F) = -K_f_ t	R^2^	0.588	0.521
Higuchi	F = K_h_ t^1/2^	R^2^	0.782	0.709
Weibull	Ln [−ln (1-F)] = −βln t_d_ + βln t	R^2^	0.973	0.988
Korsmeyer-Peppas	$\frac{F}{F_{\infty }}=$ K_k p_$.t^{n}$	k_kp_	38.318	11.307
		n	0.211	0.209
		R^2^	0.965	0.916

Note: K_0_, K_f_, K_h_, are the related drug release constant, t_d_ is time scale of the process, and F is the drug release rate at time t.

### 3.3 In-vitro cytotoxicity

**Fig. 6:**
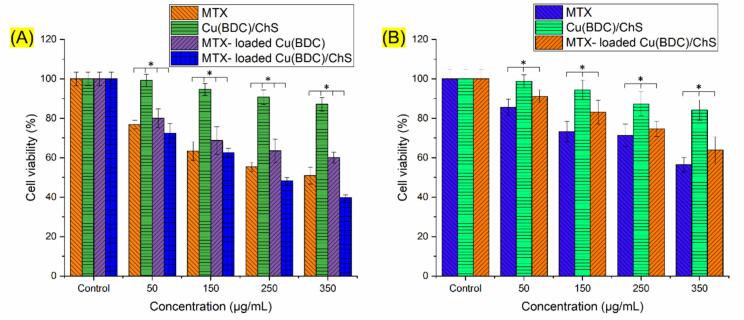
The result of MTT analysis at different concentrations of MTX, Cu(BDC)/ChS, MTX-loaded Cu(BDC) and MTX-loaded Cu(BDC)/ChS after 48 h incubation against MCF-7 (A) and HUVEC (B) cells. Statistically significant differences were determined using one-way ANOVA (*n* = 3, ^⁎^*P* < 0.05).

The cytotoxicity of the synthesized materials was evaluated using MTT assays. As shown in Fig. 6A, cytotoxic effects on MCF-7 cells were influenced by incubation concentration; higher dosages resulted in increased cytotoxicity. At equivalent concentrations of MTX, the cytotoxicity of MTX-loaded Cu(BDC)/ChS was significantly greater than that of free MTX. The IC50 values were approximately ∼250 μg/mL for MTX-loaded Cu(BDC)/ChS after a 48-h incubation period. Additionally, the cytotoxicity analysis of MTX-loaded Cu(BDC) (without ChS) showed lower toxicity compared to free MTX at equivalent concentrations, which is likely due to the sustained release of MTX from the Cu(BDC) framework, reducing the immediate intracellular drug concentration and thereby attenuating acute toxicity. This observation can confirm the passive targeting role of the Cu(BDC) carrier. In contrast, MTX-loaded Cu(BDC)/ChS exhibited substantially higher cytotoxicity than MTX-loaded Cu(BDC), highlighting the active targeting effect of ChS through CD44-mediated uptake. The enhanced cytotoxicity observed with MTX-loaded Cu(BDC)/ChS can be attributed to several factors: primarily, its positive charge improves interaction with negatively charged cellular constituents like nucleic acids, thus assisting better cellular uptake (Darvishi et al., 2024). Furthermore, the CD44-mediated feature of ChS as a result of its amide bonds can further enhance this interaction, promoting effective binding upon cellular entry, a crucial factor for cancer therapies aimed at localized tumor accumulation (Azimijou et al., 2024). The enhanced permeability and retention (EPR) effect is particularly relevant for ChS-modified systems due to the leaky blood vessels characteristic of tumor tissues resulting from rapid angiogenesis; this allows nanomedicine to accumulate within tumor microenvironments, whereas normal vessels restrict permeability (Mohammadi et al., 2024).

To assess the selectivity of the Cu(BDC)/ChS nanocomposite, MTT assays were also performed on non-cancerous HUVEC cells (Fig. 6B). The blank Cu(BDC)/ChS demonstrated good cytocompatibility, maintaining cell viability above 85 % even at a concentration of 350 μg/mL after a 48-h incubation period for both MCF-7 and HUVEC cells. The results indicated substantially lower cytotoxicity for MTX-loaded Cu(BDC)/ChS in HUVEC cells compared to MCF-7 cancer cells at equivalent concentrations (e.g., >74 % viability in HUVEC vs. ∼48 % in MCF-7 at 250 μg/mL). As shown in Fig. 6, the MTX-loaded Cu(BDC)/ChS formulation exhibited significantly higher cytotoxicity than free MTX, especially at concentrations of 250 μg/mL (*P* < 0.05). This improvement can be attributed to the presence of ChS, which facilitates CD44-mediated uptake, leading to more efficient intracellular drug accumulation. In contrast, the blank Cu(BDC)/ChS nanocarrier displayed minimal toxicity, indicating the biocompatibility of the carrier itself. These findings confirm that the designed formulation provides a statistically and functionally significant therapeutic advantage over conventional MTX delivery. This observation highlights the selective cytotoxic effect toward CD44-overexpressing cancer cells and supports the active targeting functionality of ChS in the nanocomposite. The minimal effect on normal cells also confirms the cytocompatibility profile of the Cu(BDC)/ChS. These findings support the hypothesis that the enhanced cytotoxicity in MCF-7 cells is due to receptor-mediated uptake via CD44, which is overexpressed in different cancer cell types but minimally present in normal endothelial cells. Thus, the Cu(BDC)/ChS system demonstrates promising tumor selectivity without causing significant damage to healthy cells. Overall, these findings suggest that Cu(BDC)/ChS could serve as a promising platform for an anticancer DDS.

### 3.4 Cellular apoptosis studies

**Fig. 7:**
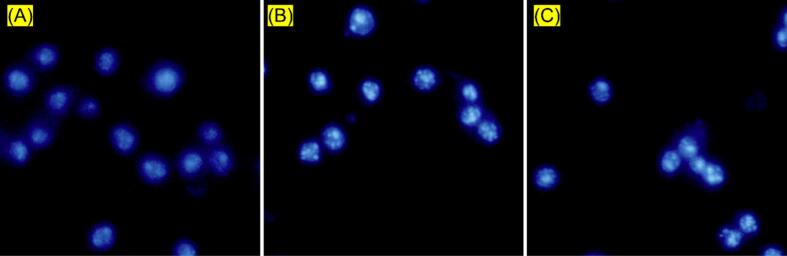
Fluorescence microscopy images of MCF-7 cells stained with DAPI after treatment with MTX-loaded Cu(BDC) (A), MTX (B), and MTX-loaded Cu(BDC)/ChS (C) at 37 °C for 24 h. Bright blue fluorescence indicates chromatin condensation and nuclear fragmentation, characteristic of apoptosis. (For interpretation of the references to colour in this figure legend, the reader is referred to the web version of this article.)

DAPI staining was utilized alongside fluorescence imaging techniques to identify cell necrosis and apoptosis based on its affinity for binding to cell nuclei (Fig. 7). MCF-7 cells treated with MTX-loaded Cu(BDC)/ChS exhibited significantly reduced viability compared to controls, accompanied by increased blue fluorescence indicative of nuclear fragmentation and chromatin condensation. In contrast, the weaker blue fluorescence observed for MTX-loaded Cu(BDC) compared to free MTX is likely due to the absence of the ChS targeting ligand; lacking CD44-mediated uptake, Cu(BDC)/MTX shows lower cellular internalization and consequently reduced apoptosis induction, which aligns with its lower cytotoxicity observed in the MTT analysis. On the other hand, fluorescence imaging related to MTX excitation confirmed that apoptosis was significantly enhanced when using MTX-loaded Cu(BDC)/ChS, indicating effective transport into cancer cells potentially evading multidrug resistance (MDR)-associated efflux mechanisms, often stated as “stealth” endocytosis. Moreover, ChS within the architecture promotes apoptosis initiation while preserving MTX integrity during delivery, supporting findings from both MTT assays and apoptosis analyses regarding successful internalization by target cells. Utilizing active targeting strategies through nanomedicine (i.e.e, CD44 receptor) for anticancer drug delivery shows promise in addressing MDR (Huang et al., 2016; Kazeminava et al., 2024). Receptor-mediated nanomedicine presents an innovative tactic to combat MDR by accelerating targeted endocytosis within cancerous cells (Lin and Lee, 2018; Poursadegh et al., 2024b). These results indicate that Cu(BDC)/ChS holds significant potential as an active targeting drug carrier aimed at improving cancer therapy outcomes.

### 3.5 Comparative studies

In comparison with conventional MTX delivery platforms, the Cu(BDC)/ChS system provides multiple advantages. While polymeric nanoparticles and liposomes offer encapsulation, they often lack precise pH-responsiveness and active targeting capabilities (Verkhovskii et al., 2023). Dendrimers and micelles provide structural versatility but may induce immunogenicity or suffer from rapid clearance (Rawding et al., 2022). In addition to these advantages over conventional polymeric or dendrimeric DDSs, the Cu(BDC)/ChS platform also shows superiority when compared to other MOF-based systems such as ZIF-8, MIL-101, or UiO-66. These MOFs, while useful for drug encapsulation, often lack active targeting ligands and rely solely on passive accumulation. MOF-based systems, particularly copper- and zinc-based ones, have been investigated for anticancer drug loading; however, their targeting capacity remains limited unless further functionalized (Sadiq et al., 2024). For example, recent work reported MTX loading on gelatin/Cu-MOF hybrids with ∼55 % loading capacity and modest release control, with notable leakage even under neutral conditions (Nezhad-Mokhtari et al., 2019). Similarly, MIL-101 and ZIF-8-based carriers release over 40 % of their payload at pH 7.4, increasing the risk of off-target toxicity (Gu et al., 2024; Moharramnejad et al., 2023). In this study, the Cu(BDC)/ChS system achieved a good MTX loading efficiency (∼70.8 %) compared to typical Cu-MOF hybrids (∼55%), and showed a markedly selective pH-responsive release profile (∼70 % at pH 4.5 vs. <20 % at pH 7.4). Furthermore, our MTT results demonstrated that MTX-loaded Cu(BDC)/ChS induced significantly greater cytotoxicity in MCF-7 cells (IC50 ≈ 250 μg/mL) than either free MTX or MTX-loaded Cu(BDC), while exhibiting much lower toxicity toward normal HUVEC cells (>74% viability at 250 μg/mL). In contrast, MTX-loaded Cu(BDC) showed lower cytotoxicity than free MTX, indicating only passive targeting via sustained release, whereas Cu(BDC)/ChS provided an additional active targeting effect via CD44-mediated uptake. Blank Cu(BDC)/ChS samples maintained >85 % cell viability, confirming their inherent biocompatibility. The present system distinguishes itself by in-situ incorporating ChS, a known CD44 ligand, which promotes receptor-mediated endocytosis. Moreover, the pH-sensitive degradation of Cu(BDC) in acidic tumor microenvironments ensures site-specific release, reducing premature drug leakage in circulation. Collectively, these experimental results provide direct evidence of the superior design and therapeutic promise of Cu(BDC)/ChS as a tumor-targeted chemotherapy platform.

While the in vitro results of the Cu(BDC)/ChS nanocomposite are highly promising in terms of selective cytotoxicity, drug release, and cancer cell targeting, it is important to acknowledge that in vitro conditions cannot fully replicate the complexity of living systems. Further in vivo studies are essential to evaluate systemic biodistribution, pharmacokinetics, tumor accumulation via the enhanced permeability and retention (EPR) effect, and potential immunogenic or off-target effects. Such studies will be crucial to assess the real-world therapeutic viability and safety profile of this MOF-based nanocarrier and validate its translational applicability.

## 4 Conclusion

In summary, the Cu(BDC)/ChS nanomedicine was successfully synthesized using a one-pot approach and characterized through different analytical methods, such as FT-IR, XRD, TGA, SEM, TEM, DLS, and ZP. The neoteric integration of Cu(BDC) with ChS resulted in a passive and active targeted DDS that demonstrated pH-controlled release profiles and enhanced drug efficacy. In-vitro studies showed that MTX release rates ∼70 % after 72 h in acidic conditions mimicking tumor environments (pH 4.5, 41 °C), compared to ∼20 % in physiological conditions (pH 7.4, 37 °C). The MTX-loaded Cu(BDC)/ChS nanocomposite exhibited significant cytotoxic effects on MCF-7 cells with an IC50 of ∼250 μg/mL. These results indicate that the combination of MOF structures with receptor modifications in DDSs can significantly improve cancer therapy outcomes. However, it must be emphasized that these conclusions are based solely on in vitro data. Although the results are promising, it should be mentioned that further comprehensive in vivo studies are essential to validate the therapeutic viability of the Cu(BDC)/ChS system in terms of biodistribution, tumor targeting efficiency, pharmacokinetics, and long-term safety in biological systems. Additionally, exploring the incorporation of other compounds or phytochemicals into the Cu(BDC)/ChS framework could further enhance therapeutic effectiveness. Therefore, while the Cu(BDC)/ChS nanocomposite shows substantial potential as a tumor-targeted drug delivery platform, its clinical translation requires systematic in vivo validation in future studies.

## CRediT authorship contribution statement

**Siamak Javanbakht:** Writing – original draft, Visualization, Validation, Software, Project administration, Methodology, Investigation, Formal analysis, Data curation, Conceptualization. **Reza Mohammadi:** Writing – review & editing, Validation, Supervision, Resources.

## Declaration of competing interest

The authors declare that they have no known competing financial interests or personal relationships that could have appeared to influence the work reported in this paper.

## Data Availability

Data will be made available on request.
